# Molecular Identification and Taxonomic Implication of Herbal Species in Genus *Corydalis* (Papaveraceae)

**DOI:** 10.3390/molecules23061393

**Published:** 2018-06-08

**Authors:** Lu Jiang, Meihui Li, Fengxi Zhao, Shanshan Chu, Liangping Zha, Tao Xu, Huasheng Peng, Wei Zhang

**Affiliations:** 1 Marine College, Shandong University, Weihai 264209, China; lujiang5696@126.com (L.J.); limeihuisddx0708@126.com (M.L.); zfxwaxs@163.com (F.Z.); 2 College of Pharmacy, Anhui University of Chinese Medicine, Hefei 230012, China; cshan0916@126.com (S.C.); chaliangping520@126.com (L.Z.); xutaojy@126.com (T.X.); 3 Institute of Traditional Chinese Medicine Resources Protection and Development, Anhui Academy of Chinese Medicine, Hefei 230012, China

**Keywords:** *Corydalis* DC, *Corydalis yanhusuo*, *Corydalis decumbens*, DNA barcoding, species identification, traditional Chinese medicine

## Abstract

Many species of *Corydalis* (Papaveraceae) have been used as medicinal plants in East Asia, and the most well-known species are *Corydalis yanhusuo* and *C. decumbens* in the Pharmacopoeia of China. However, authentication of these species remains problematic because of their high morphological variation. Here, we selected 14 closely related species and five genomic regions (chloroplast: *mat*K, *trn*G, *rbc*L, *psb*A-*trn*H; nuclear: ITS) to explore the utility of DNA barcoding for authenticating these herbs. In addition, the Poisson tree process (PTP) and automatic barcode gap discovery (ABGD) were also used and compared with DNA barcoding. Our results showed that the ITS region was not suitable for molecular analysis because of its heterogeneous nature in *Corydalis*. In contrast, *mat*K was an ideal region for species identification because all species could be resolved when *mat*K was used along with the other three chloroplast regions. We found that at least five traditional identified species were lumped into one genetic species by ABGD and PTP methods; thus, highlighting the overestimation of species diversity using the morphological approach. In conclusion, our first attempt of molecular analysis of *Corydalis* herbs presented here successfully resolved the identification of medicinal species and encouraged their taxonomic re-assessment.

## 1. Introduction

*Corydalis* DC., comprising ca. 465 species with a variety of morphological and taxonomical diversity, is the largest genus in the family Papaveraceae. It is mainly distributed in the north temperate region, with China possessing 80% of its species diversity and 56% species endemism [1]. Species of this genus are often characterized by their showy spurs and thus have some ornamental value. More importantly, some species with tuberous roots, especially those belonging to the Section *Corydalis* and Section *Duplotuber*, are often used as traditional medicine in East Asia. For example, *C. yanhusuo* W. T. Wang ex Z. Y. Su et C. Y. Wu and *C. decumbens* (Thunb.) Pers. are most famous medicinal plants recorded in the Pharmacopoeia of China and are used for promoting blood circulation and stopping pain in many prescriptions [2]. Other commonly used species of herbal medicines include *C. turtschaninovii* Bess., *C. ternata* (Nakai) Nakai, *C. fumariifolia* Maxim., *C. ambigua* Cham. et Schltdl., and *C. humosa* Migo, which have been recorded in the Pharmacopoeia of Japan and Korea and are also commonly used in some local areas of China [3,4]. A severe problem with these herbal species is that many erroneous substitutes and adulterants are traded in the medicinal materials market, mainly because of the misidentification of species with similar morphological features or complex morphological variation. Although morphological, anatomical, and chemical profiling methods have been used to authenticate these species [5,6,7,8,9], none of these methods can easily identify the target species, especially for processed products. Although molecular phylogenetic studies referring to *Corydalis* species have been conducted by many researchers [10,11,12,13,14], most provided some basic frameworks of taxonomy at a higher level of tribes and subgenus. Therefore, until recently few studies have been conducted for closely related species, and it is still unclear if the herbal species of *Corydalis* can be identified using the DNA barcoding technique.

Recent years have witnessed an increasing number of molecular approaches for species delimitation [15]. Because of the ease of sequencing using universal primers, single-locus methods for species delineation are still a more practical way to delimit large-scale datasets compared with methods based on multi-locus or genome-wide data. In addition to conventional DNA barcoding, we selected two other distinct single-locus methods for comparison. One is a genetic distance-based approach, the Automatic Barcode Gap Discovery (ABGD) method [16]. It is a fast, simple method that sorts sequences into hypothetical species based on barcode gaps whenever intraspecific genetic distances are smaller than that among organisms from different species. The other is a tree-based method, the Poisson tree process (PTP) [17]. This method models specimen in terms of the number of substitutions and has been shown to be one of the most robust methods for preliminary species delimitation [17,18].

Here, we performed multiple molecular analyses (DNA barcoding, ABGD, PTP) on nuclear internal transcribed spacer (ITS) and four chloroplast DNA (cpDNA) regions (*rbc*L, *trn*H-*psb*A, *mat*K, *trn*G), which have been widely used in plant DNA barcoding, in order to (1) clarify how well these markers can distinguish the commonly used herbal species of *Corydalis*, (2) ascertain the association between molecular phylogeny and morphological features, and (3) detect the number of candidate species objectively and provide some insights into the taxonomy of these taxa.

## 2. Results

### 2.1. Sequence Analyses of ITS Region

The ITS region was amplified using two pairs of candidate primers. The full length of ITS was amplified by ITS4/ITS5B, and its PCR amplification success rate and sequencing success rate was 61.9% and 28.6%, respectively. Alternatively, the ITS2 region was amplified by ITS2F/ITS3R with a 100% PCR amplification success rate and a 69.10% sequencing success rate. Notably, some heterozygous sequences were detected among the chromatogram files of ITS direct sequencing. The five PCR amplification products were cloned and 46 sequences were obtained and used to construct a Neighbor Joining (NJ) tree. Our results showed that the sequences from the same individuals were divided into two distinct clades with high support. Obviously, these sequences from the same individual or species failed to form a monophyletic group (Figure S1).

### 2.2. DNA Barcoding Analyses Using Plastid Regions

#### 2.2.1. Plastid Region Test for Screening Efficient DNA Barcodes

A total of 57 samples and 228 sequences were successfully PCR amplified and sequenced with high quality. The lengths of the aligned DNA fragments of *mat*K (K), *trnG* (G), *trn*H*-psb*A (H) and *rbc*L (L) were 859 bp, 730 bp, 468 bp, and 688 bp, respectively. We found that the *mat*K region generated the largest number of variable characters (151) and had the highest number of both parsimony information characters (117) and species identification rate (78.57%). Although *trnH-psbA* provided the largest rate of variable characters (19.44%) and parsimony information characters (14.32%), it had the lowest species identification rate (42.86%). To obtain a higher efficiency for species identification, the combinations of *mat*K and different DNA regions were compared and assessed. The results showed that the combination of K + G resolved 85.71% species (Table 1, Figure S1b), which had the highest rate of species identification among the combinations of any two loci. The combination of K + G + H generated the largest rate of variable characters (15.41%) and parsimony information characters (11.62%), as well as the highest rate of species identification (92.86%) among the combinations of any three loci. In contrast, when four cpDNA were combined, all species were successfully identified (Table 1). 

#### 2.2.2. Barcoding Gap in Sect. *Corydalis*

**Figure 1:**
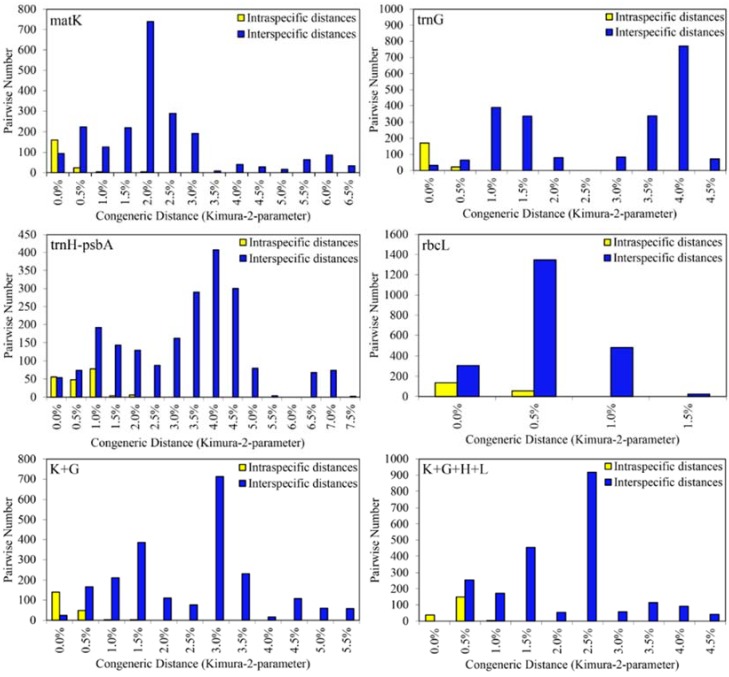
Intra- and interspecific genetic divergences in Sect. *Corydalis*.

The 49 individuals of 11 species generated 192 intraspecific and 2160 interspecific pairwise comparisons. The barcoding gap between intra- and interspecific distance was determined by graphing the distribution of variation in kimura-2-parameter distances. Both the minimum intra- and interspecific distance was 0 for all loci and their combinations. The *trn*H*-psb*A had the highest interspecific divergence (7.5%), followed by *mat*K (6.5%)*.* Correspondingly, *mat*K, *trn*H*-psb*A, and K + H had the maximum intraspecific divergence (2.0%), followed by K + G (1.5%)*.* We did not find barcoding gaps in any of the single- or combined barcodes (Figure 1). Furthermore, *trn*H*-psb*A and *mat*K exhibited more obvious difference between intra- and the interspecific divergence.

#### 2.2.3. Species Discrimination

**Figure 2:**
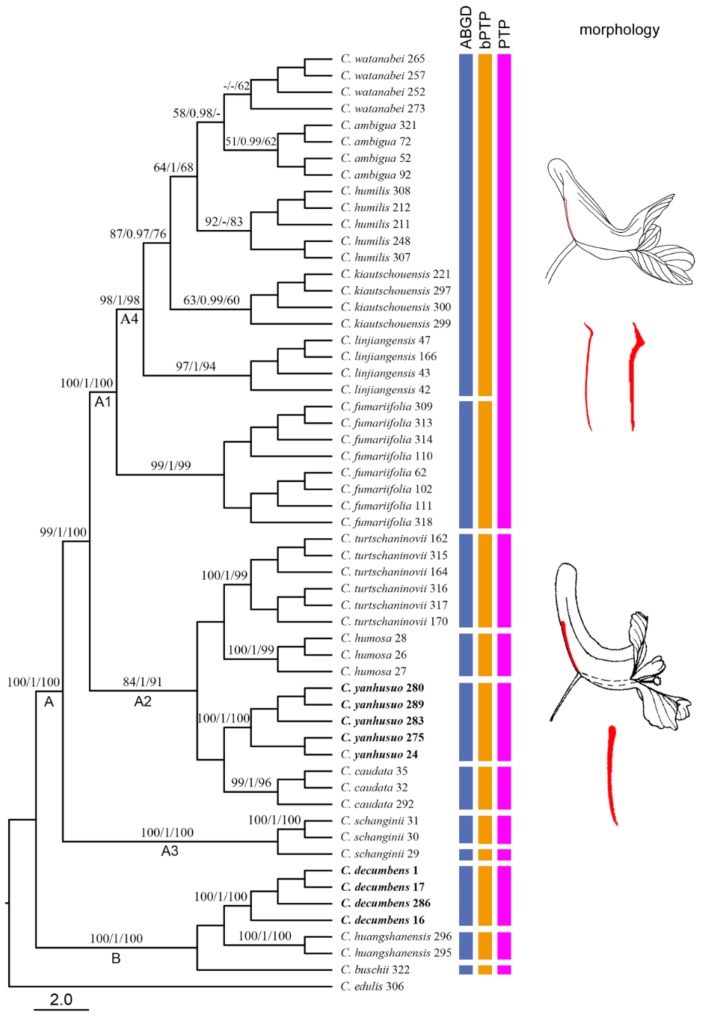
Neighbor Joining (NJ) tree based on the four combined chloroplast loci (*mat*K, *trn*G, *rbc*L, *trn*H*-psb*A). The numbers on the branches indicate the support value of Maximum Likelihood (ML)/Bayesian Inference (BI)/NJ (>50%). The numbers following a species name represent the numbers of individuals. Cluster A represents Sect. *Corydalis*. Cluster B represents Sect. *Duplotuber*. The column bar indicates the putative species identified by Automatic Barcode Gap Discovery (ABGD) and Poisson tree process (PTP). Acute to acuminate nectary corresponds to Cluster A1. Obtuse nectary corresponds to Cluster A2 and A3.

Distance analysis performed in TaxonDNA (http://taxondna.sourceforge.net/) showed that the *trnG* region had the highest rate of successful species discrimination (83.67%), followed by *mat*K (73.46%). When all four loci were combined, all species could be successfully identified (Figure S2). The monophyletic test based on the NJ tree showed that when the four loci were used in combination, all species with multiple individuals were successfully resolved as monophyletic clusters (Figure 2). In these clades, the target herbal species were successfully identified because their individuals were clustered together into a monophyletic group with strong support, which separated them from their closest relatives. Notably, the single *mat*K region, identified 11 of 14 species and all the herbal species based on a monophylogenetic test, and had the highest discriminatory power among the single four loci (Table 1).

### 2.3. Molecular Delimitation and Classical Taxonomy

In the combined cpDNA NJ tree, the 56 individuals formed 14 species-specific clusters and each cluster corresponded to a taxonomic group recognized by previous authors (Figure 2). All the 14 clusters formed two main clades with high support, which corresponded to the root-bulbed Sect. *Corydalis* and the root-tubered Sect. *Duplotuber* (Figure 2 A, B). On the whole, the species relationships displayed in the NJ tree can be interpreted using the shape of nectary and the length ratio of nectary to spur; i.e., the species with acute or acuminate nectary were clustered together into one clade (Figure 2 A1) and it was sister to those species with an obtuse nectary 1/3-2/3 as long as the spur (Figure 2 A2); *C. schanginii* Regel, with an obtuse nectary being less than 1/4 as long as the spur, was the base clade (Figure 2 A3) of the two former sister groups.

**Figure 3:**
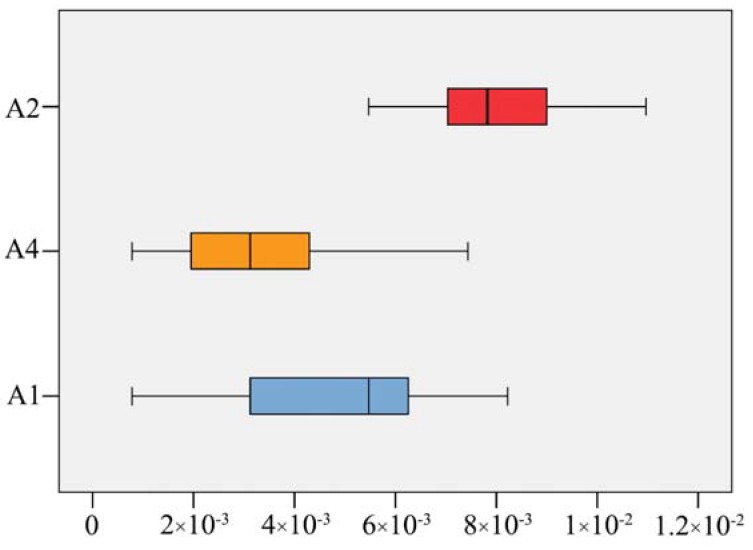
Box-plot representing interspecific kimura-2-parameter pairwise distances (x-axes) among different groups (y-axes). A2 includes species *C. turtschaninovii*, *C. humosa*, *C. yanhusuo,* and *C. caudata*. A4 includes species *C. watanabei*, *C. ambigua*, *C. humilis*, *C. kiautschouensis,* and *C. linjiangensis*, and A1 includes A4 + *C. fumariifolia* (See Figure 2).

In clade A2, each monophyletic group corresponded well with the species-partition generated from ABGD, PTP, and bPTP*.* However, it was not the case in the other clades. In clade A1, the five or six prior recognized species were lumped into one species-partition. By comparison, in clade A3 individuals of *C. schanginii* were split into two species-partitions (Figure 2). We compared the genetic distance within the clades A1, A2, and A4, and found that the interspecific kimura-2-parameter pairwise distances for A1 and A4 were smaller than those for A2 (Figure 3). Furthermore, using an independent sample *t*-test, we found that the interspecific pairwise distances for A1 vs. A2 and A4 vs. A2 were significantly different (*p* < 0.05).

## 3. Discussion

### 3.1. Multi-Copy Nature of ITS in Corydalis

The ITS region of rDNA is the most widely used phylogenetic marker and has contributed greatly to plant phylogeny. Recently, ITS2 has been suggested as one of the standard core barcodes and the only nuclear barcode available for the identification of seed plants [19]. This region has also been used in previous studies of *Corydalis* species in the context of constructing phylogenies at higher taxonomic levels [10,11,13]. However, up to now no study has reported the multi-copy nature of ITS in *Corydalis* or at its higher Papaveraceae level. We found inconsistent phylogenies between the nuclear ITS and chloroplast trees in Sect. *Corydalis* of *Corydalis* and multiple heterozygous ITS copies after the ITS PCR amplifications were cloned and the sequences were used to build the tree. Thus, our results could explain the inconsistent phylogenetic position of some species in previous phylogenetic inferences from ITS and highlight that related studies of *Corydalis* using ITS should be carried out with caution.

### 3.2. matK Is a Promising DNA Barcode for Corydalis

In plant DNA barcoding, cpDNA has attracted more attention not only because of the slow substitution rates of mitochondrial DNA, but also because of the technical difficulties in the use of low-copy nuclear genes. In this study, four candidate cpDNA barcodes were evaluated because of the heterozygosity of the nrITS in the genus *Corydalis*. Our results showed that the *matK* region (matK1166-matK192), suggested by Pérez-Gutiérrez et al. [10], provided the highest sequence variability and species resolution among the four commonly used barcoding regions. This single-locus barcode had high efficiency in discriminating closely related species in *Corydalis* and worked well for some specific purposes, such as the identification of herbal species. There have been concerns about the difficulties of amplification and sequencing associated with *matK* used in some groups, which resulted from low primer universality. However, in the present study, the PCR and sequencing success rates were both 100% for the *matK* region. Moreover, *matK* had 100% species discriminating power when combined with the other three regions in this study. Taken together, these results suggested that *matK* was a promising DNA barcode in *Corydalis*.

### 3.3. Taxonomic Implications of Molecular Identification in Corydalis

This study presents the first phylogenetic study of the most closely related Sect. *Corydalis* and Sect. *Duplotuber*. We noticed that our results of molecular phylogenetic analyses not only successfully distinguished the closely related species in *Corydalis* but also provided some new insights into their taxonomy. *C. fumariifolia* and *C. ambigua* are very similar in morphology and have always been considered as a same species of different variants, subspecies or forms [5,11]. However, our phylogenetic analyses strongly support a monophyletic *C. fumariifolia* and *C. ambigua* but do not support their sister relationship. This finding firstly confirmed their distinct species status using molecular information. Although our phylogenetic analyses showed that all the traditional recognized species exhibited reciprocal monophyly and thus could be successfully identified, it does not mean they were without problems. The species status in clade A4 (Figure 2) has been confused for a long time [5]. Although they recently have been recognized as five separate species, the paucity of morphological diagnostic characters, together with their overlapping and convergent nature lead to confused taxonomy. Our alternative species delimitation approaches (ABGD and bPTP) showed that they all belonged to a single species. In addition, genetic distances between these species were much smaller than those between their relatives in clade A2. Taken together, we propose that *C. watanabei* Kitagawa, *C. ambigua* Cham. & Schltdl., *C. humilis* Oh et Kim, *C. kiautschouensis* V. Poelln., and *C. linjiangensis* Z. Y. Su ex Liden should be regarded as a single species. In a larger sense, this result indicates that monophyly should not be used as the sole criterion for species delimitation in phylogenetic analyses. Theoretically, even the tiniest subdivision of a species could be made diagnosable if the molecular markers have adequate discriminatory power. As a result, an unwarranted explosion of species numbers may occur through the splitting of existing species, rather than the identification of new species. In this case, it would be helpful to provide a divergence threshold as a reference point for determining a species boundary [20]. In summary, these findings highlighted the necessity of using different molecular methods to clear the complex taxonomic classification of *Corydalis*.

## 4. Materials and Methods

### 4.1. Taxonomic Sampling

A total of 56 individuals from 53 different populations, representing 11 species of Sect. *Corydalis* (68.75%) and three species of Sect. *Duplotuber* (75%), were collected from the wild from 2014 to 2017 at different areas of their distribution (Table S1). Moreover, *C. edulis* Maxim. was selected as the outgroup according to a previous phylogenetic study [21]. All voucher samples were authenticated by Prof. Dr. Huasheng Peng and deposited in the College of Pharmacy, Anhui University of Chinese Medicine, Hefei, China.

### 4.2. DNA Extraction, Amplification, and Sequencing

Total genomic DNA was extracted from 20 mg of leaf tissue dried in silica gel, using the modified CTAB protocol [22]. The ITS2 region and the four plastid fragments (*rbc*L, *trn*H-*psb*A, *mat*K, and *trn*G) were amplified using the universal primers suggested in previous studies [11,23,24,25,26,27,28] (Table S2). A few DNA templates with impurity were purified with a TIANGEN DNA purification kit (Tiangen Biotech, Beijing, China). The PCR reactions were performed in a volume of 25 μL, including 40–100 ng of template DNA, 2.5 μL 2.5 mM each NTP, 2.5 μL 10 × PCR buffer, 0.5 μL 10 uM of each primer, and 0.625 U Taq Polymerase. The PCR programs for all five loci were as follows: 94 °C for 4 min; 35 cycles at 94 °C for 30 s, 53 °C for 30 s, 72 °C for 1.5 min; followed by a single cycle at 72 °C for 10 min. The PCR products were sequenced on an ABI 3730XL sequencer (Applied Biosystems, Foster City, CA, USA) using the PCR primers. In addition, five PCR products of the ITS2 regions with confusing relationships in the cpDNA tree, were cloned with the pUCm-T carrier system [29]. More than eight colonies per individual were selected and sequenced with the primers M13-(-48).

### 4.3. Statistical Analysis

The PCR amplification rate and sequencing success rate were calculated as the ratios between the successfully amplified or sequenced individuals and all individuals amplified or sequenced. All sequences were aligned and adjusted manually using BioEdit version 7.2.2 [30]. All of the cpDNA fragments were combined using Clustal X 1.8 [31]. Barcoding gaps were evaluated using the genetic divergences between intra- and interspecific samples and performed through TaxonDNA 1.7.7 [32]. The Neighbor-Joining (NJ) trees were constructed using MEGA 6.0 [33] based on the Kimura-2-parameter model, and the branch support values were evaluated with 1000 bootstrap replications. The pairwise distances were calculated by MEGA 6.0 and used to construct box-plot by SPSS 24 (SPSS, Chicago, IL, USA). The phylogenetic characters of each locus and their combinations, including the variable characters, parsimony information characters, consistency index, and retention index were calculated through PAUP 4.0 [34]. The GTR + G model, calculated in jModeltest2 [35], was selected for maximum-likelihood (ML) and Bayesian inference (BI) analysis. The ML analysis was performed in RAxML [36] with 1000 bootstrap replications. The BI tree was implemented in MrBayes 3.2.6 [37]. Four Monte Carlo Markov chains were run from random trees for 10,000,000 generations and sampled every 1000 generations. The first 2500 generations (25%) were discarded as “burn-in” from each run.

The ABGD method is available at webpage http://wwwabi.snv.jussieu.fr/public/abgd/abgdweb.html. 

The combined cpDNA dataset was used as the input file [16]. With the Kimura-2-parameter model, a range of different settings resulted in the same number of initial partitions. Therefore, we set the parameters as follows: *P*_min_ = 0.0008, *P*_max_ = 0.007, Steps = 50, *X* = 1, Nb bins = 20 (Figure S3). PTP and bPTP analyses were implemented on the bPTP web server (http://species.h-its.org/). The rooted-trees from ML and BI analyses were input without ultrametrics. We ran PTP and bPTP analyses with default values after removing the outgroup and the ML optimal algorithm tree was adopted as the output file [17].

## Figures and Tables

**Table 1:** Statistics of phylogenetic analyses from the four DNA loci and their combinations.

Statistic	K	G	L	H	K + G	K + G + H	K + G + H + L
Amplification success rate (%)	100	100	100	100			
Sequencing success rate (%)	100	100	100	100			
Length range (bp)	834–849	692–681	688	333–456	1526–1552	1872–1888	2562–2676
Aligned length (bp)	859	730	688	468	1589	2057	2745
No. of variable characters (%)	151 (17.58)	75 (10.27)	37 (5.38)	91 (19.44)	226 (14.22)	317 (15.41)	354 (12.90)
No. of parsimony information characters (%)	117 (13.62)	55 (7.53)	28 (4.07)	67 (14.32)	172 (10.82)	239 (11.62)	267 (9.73)
CI	0.9257	0.9011	0.7872	0.8000	0.9071	0.8544	0.8330
RI	0.9812	0.9803	0.8834	0.9386	0.9783	0.9623	0.9547
Species identification rate (%)	78.57	64.29	50.00	42.86	85.71	92.86	100

K, *mat*K; G, *trn*G; H, *trn*H-*psb*A; L, *rbc*L.

## Supplementary Materials

The following are available online. Figure S1: Neighbor joining (NJ) tree based on the different data sets; Figure S2: Species identification efficiency based on “Best Match”; Figure S3: Graph of ABGD web results using K80 Kimura measure of distance; Table S1: Collection details and NCBI accession numbers of samples used in this study; Table S2: The universal amplification primers used in this study.
