# Supplementary material for: Molecular Identification and Taxonomic Implication of Herbal Species in Genus Corydalis (Papaveraceae)
Source: Molecules. 2018 Jun 8;23(6):1393. doi: 10.3390/molecules23061393 (PMC6100380; doi:10.3390/molecules23061393)
Supplement: Supplementary file 1 [file molecules-23-01393-s001.pdf]

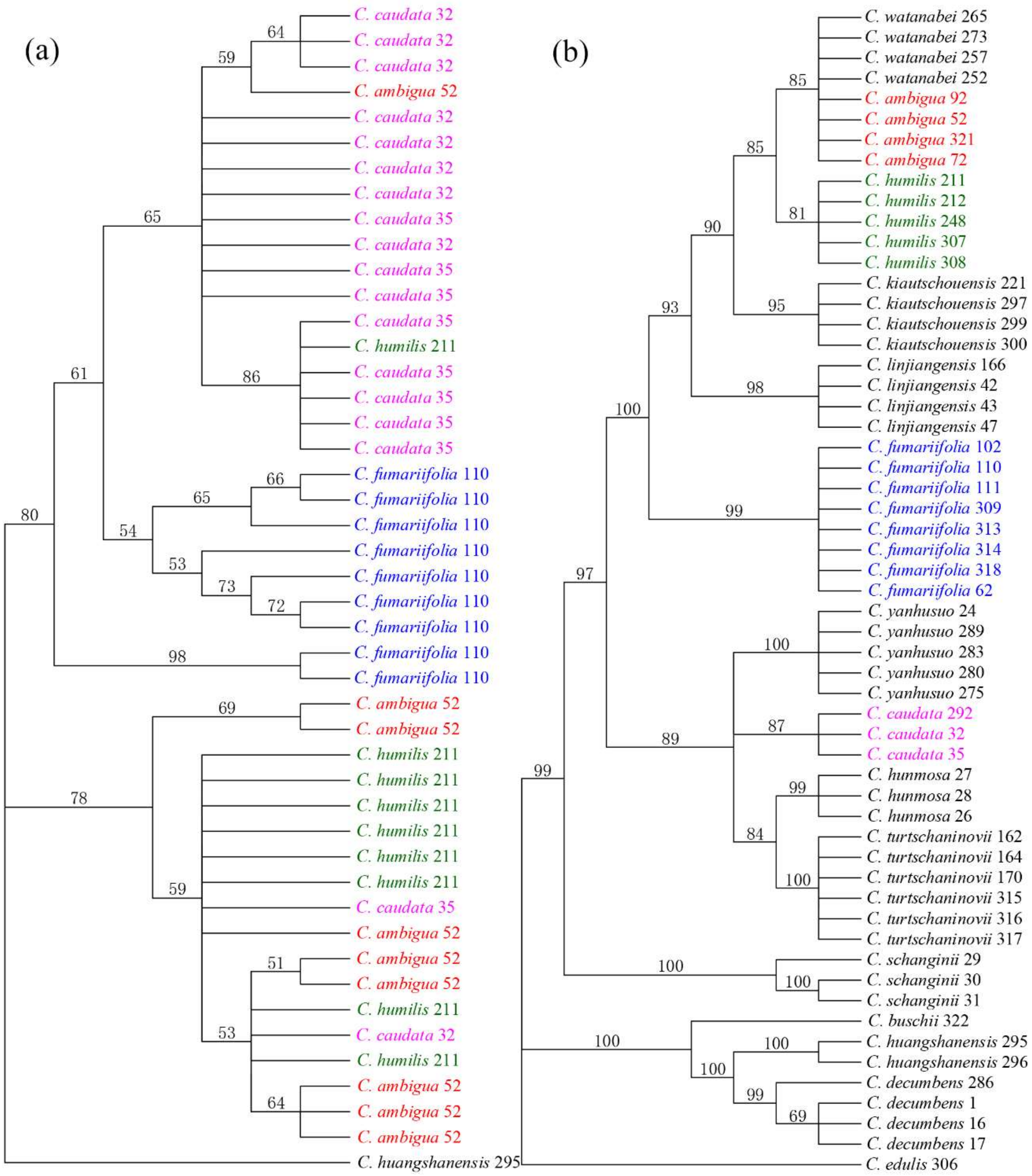

Figure S1: Neighbor joining (NJ) tree based on the different data sets.

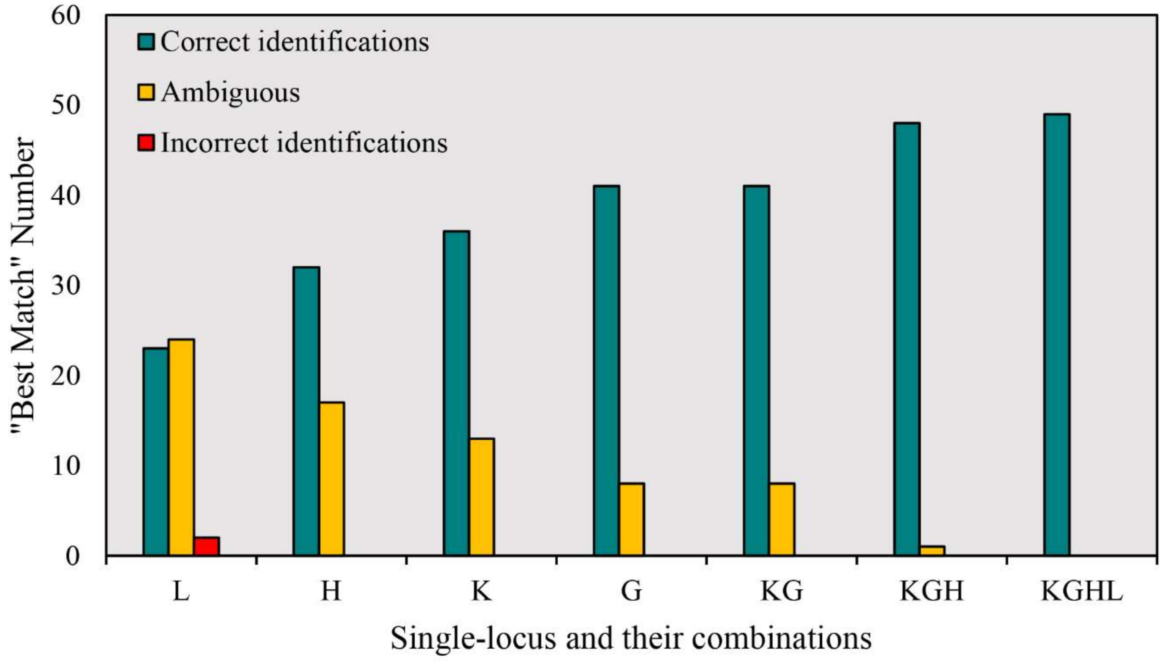

Figure S2: Species identification efficiency based on “Best Match”.

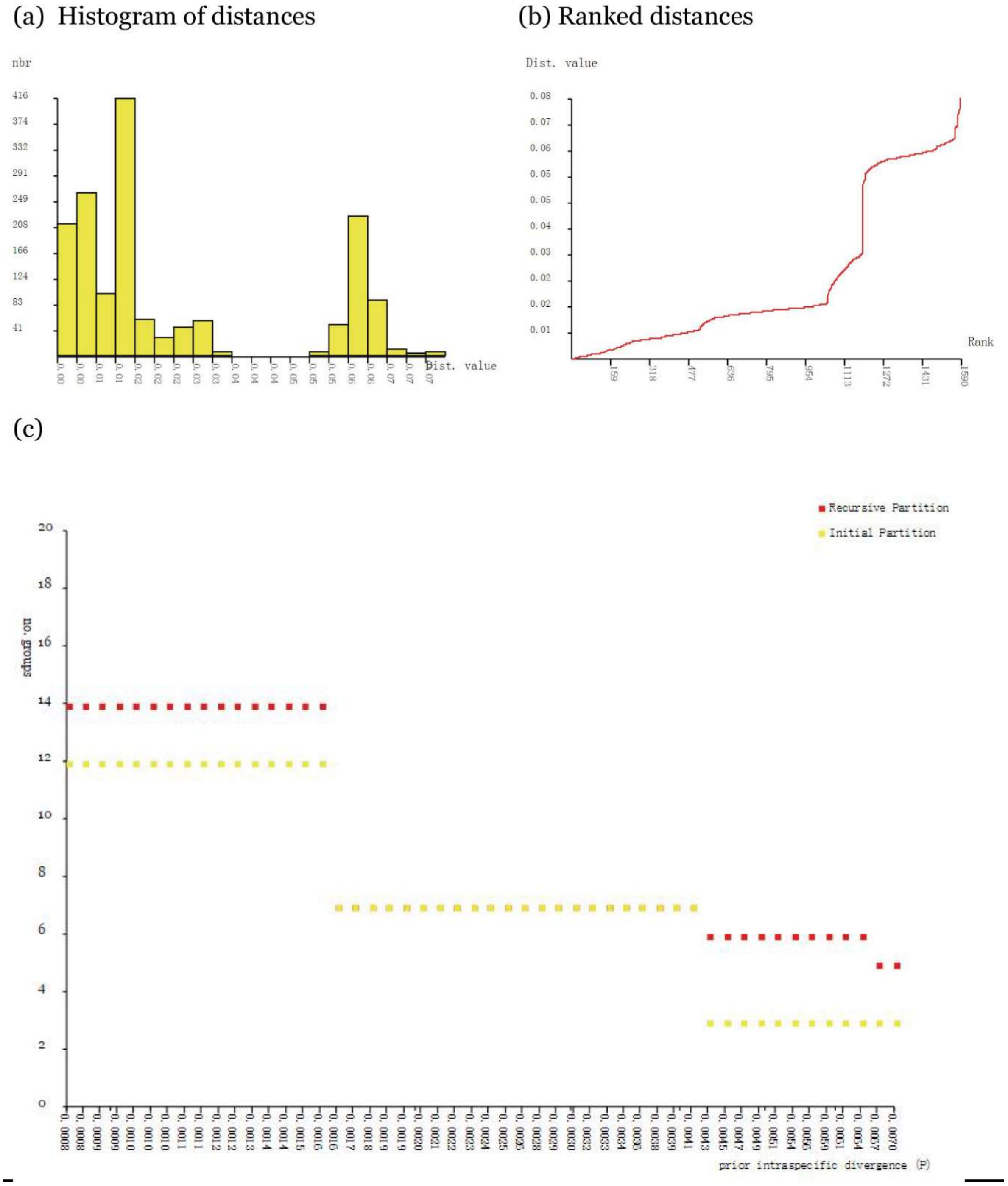

Figure S3: Graph of ABGD web results using K80 Kimura measure of distance.

Table S1: Collection details and NCBI accession numbers of samples used in this study.

Species	Locality of Collection	Longitude, Latitude and altitude	matK	psbA-trnH	rbcL	trnG
Corydalis ambigua 321	Ji'an City, China	E126°20′13.3″N41°22′42.8″H602.1m	MF573564	MF573619	MF573674	MF573729
Corydalis ambigua 52	Ji'an City, China	E126°20′14.2″N41°22′41.3″H614m	MF573565	MF573620	MF573675	MF573730
Corydalis ambigua 72	Tonghua City, China	E126°01′3.7″N41°43′15.9″H588m	MF573566	MF573621	MF573676	MF573731
Corydalis ambigua 92	Linjiang City, China	E126°45′34.5″N41°54′21.8″H656m	MF573567	MF573622	MF573677	MF573732

Corydalis buschii 322	Baishan City, China	E127°28′2.06″N42°09′34.45″ H753m	MG748615	MG748617	MG748619	MG748621
Corydalis caudata 292	Suzhou City, China	E116°52′30.75″N34°10′01.59″H78m	MF573568	MF573623	MF573678	MF573733
Corydalis caudata 32	Luoyang City, China	E111°50′15.81″N33°41′26.87″H1463m	MF573569	MF573624	MF573679	MF573734
Corydalis caudata 35	Luoyang City, China	E111°50′15.85″N33°41′25.18″H1490m	MF573570	MF573625	MF573680	MF573735
Corydalis decumbens 1	Tongling City, China	E117°56′57.86″N30°54′07.75″H186m	MF573571	MF573626	MF573681	MF573736
Corydalis decumbens 16	Chuzhou City, China	E117°33′36.09″N32°52′48.87″H59m	MF573572	MF573627	MF573682	MF573737
Corydalis decumbens 17	Ma'anshan City, China	E118°34′53.74″N31°35′42.18″H130m	MF573573	MF573628	MF573683	MF573738
Corydalis decumbens 286	Huangshan City, China	E117°31′51.58″N30°00′53.99″H468m	MF573574	MF573629	MF573684	MF573739
Corydalis edulis 306	Chuzhou City, China	E117°33′38.92″N32°52′53.84″H81m	MF573575	MF573630	MF573685	MF573740
Corydalis fumariifolia 102	Jilin City, China	E126°37′23.67″N43°25′29.81″H591m	MF573576	MF573631	MF573686	MF573741
Corydalis fumariifolia 110	Shangzhi City, China	E127°33′17.47″N45°17′44.99″H329m	MF573577	MF573632	MF573687	MF573742
Corydalis fumariifolia 111	Mudanjiang City, China	E128°54′39.1″N44°03′36.2″ H355m	MF573578	MF573633	MF573688	MF573743
Corydalis fumariifolia 309	Huinan County, China	E126°35′39.9″N42°32′7.8″H649m	MF573579	MF573634	MF573689	MF573744
Corydalis fumariifolia 313	Huinan County, China	E126°16′59.1″N42°26′3.2″H389.7m	MF573580	MF573635	MF573690	MF573745
Corydalis fumariifolia 314	Tonghua City, China	E126°35′38.08″N42°31′56.04″H620m	MF573581	MF573636	MF573691	MF573746
Corydalis fumariifolia 318	Harbin City, China	E127°33′47.19″N45°17′39.51″H353m	MF573582	MF573637	MF573692	MF573747
Corydalis fumariifolia 62	Benxi City, China	E124°53′15.92″N41°19′3.84″H1220m	MF573583	MF573638	MF573693	MF573748
Corydalis huangshanensis 295	Huangshan District, China	E118°08′50.04″N30°05′14.92″H639m	MF573584	MF573639	MF573694	MF573749
Corydalis huangshanensis 296	Huangshan District, China	E118°09′44.58″N30°03′39.03″ H600m	MF573585	MF573640	MF573695	MF573750
Corydalis humilis 211	Dongchang District, China	E125°59′24.7″N41°44′25.9″H443.9m	MF573586	MF573641	MF573696	MF573751
Corydalis humilis 212	Ji'an City, China	E125°43′16.05″N41°14′40.89″H604m	MF573587	MF573642	MF573697	MF573752
Corydalis humilis 248	Liuhe County, China	E126°10′2.4″N42°07′49.1″H591.8m	MF573588	MF573643	MF573698	MF573753
Corydalis humilis 307	Huinan County, China	E126°35′44.79″N42°32′12.95″H633m	MF573589	MF573644	MF573699	MF573754
Corydalis humilis 308	Tonghua City, China	E126°35′53.31″N42°32′7.35″H700m	MF573590	MF573645	MF573700	MF573755
Corydalis humosa 26	Song County, China	E111°50′11.38″N33°41′20.78″H1650m	MF573591	MF573646	MF573701	MF573756
Corydalis humosa 27	Song County, China	E111°50′11.38″N33°41′20.77″H1648m	MF573592	MF573647	MF573702	MF573757
Corydalis humosa 28	Song County, China	E111°50′11.37″N33°41′20.66″H1640m	MF573593	MF573648	MF573703	MF573758
Corydalis kiautschouensis 221	Benxi City, China	E125°25′10.4″N41°13′14.0″H440m	MF573594	MF573649	MF573704	MF573759
Corydalis kiautschouensis 297	Weihai City, China	E122°06′6.1″N37°28′57.8″H225m	MF573595	MF573650	MF573705	MF573760
Corydalis kiautschouensis 299	Muping district, China	E121°50′09.45″N37°12′15.61″H125m	MF573596	MF573651	MF573706	MF573761
Corydalis kiautschouensis 300	Yantai City, China	E121°50′19.5″N37°12′15.77″H119m	MF573597	MF573652	MF573707	MF573762

Corydalis linjiangensis 166	Tonghua City, China	E126°05′5.9″N41°53′56.4″H930m	MF573598	MF573653	MF573708	MF573763
Corydalis linjiangensis 42	Linjiang City, China	E126°42′6.7″N41°51′14.9″H1041m	MF573599	MF573654	MF573709	MF573764
Corydalis linjiangensis 43	Linjiang City, China	E126°45′10.0″N41°54′33.6″H710m	MF573600	MF573655	MF573710	MF573765
Corydalis linjiangensis 47	Baishan City, China	E126°40′14.27″N41°53′32.47″H819m	MF573601	MF573656	MF573711	MF573766
Corydalis schanginii 29	Aletai City, China	E88°08′58.53″N47°50′01.38″H1003m	MF573602	MF573657	MF573712	MF573767
Corydalis schanginii 30	Fuhai County, China	E87°29′04.12″N47°06′14.41″H497m	MG748616	MG748618	MG748620	MG748622
Corydalis schanginii 31	Aletai City, China	E87°20′12.13″N47°49′37.85″H992m	MF573603	MF573658	MF573713	MF573768
Corydalis turtschaninovii 162	Dongchang District, China	E126°19′45.6″N41°26′58.1″H678m	MF573604	MF573659	MF573714	MF573769
Corydalis turtschaninovii 164	Dongchang District, China	E126°20′01.49″N41°26′51.72″H711m	MF573605	MF573660	MF573715	MF573770
Corydalis turtschaninovii 170	Jilin City, China	E126°37′40.80″N43°25′27.89″H607m	MF573606	MF573661	MF573716	MF573771
Corydalis turtschaninovii 315	Huinan County, China	E126°35′42.1″N42°32′8.3″H671m	MF573607	MF573662	MF573717	MF573772
Corydalis turtschaninovii 316	Baishan City, China	E127°17′12.4″N41°30′47.0″H699m	MF573608	MF573663	MF573718	MF573773
Corydalis turtschaninovii 317	Liaoyuan City, China	E125°17′12.4″N42°23′49.6″H420m	MF573609	MF573664	MF573719	MF573774
Corydalis watanabei 252	Tonghua City, China	E126°18′24.94″N41°23′50.48″H813m	MF573610	MF573665	MF573720	MF573775
Corydalis watanabei 257	Benxi City, China	E124°53′55.7″N41°18′4.5″H822m	MF573611	MF573666	MF573721	MF573776
Corydalis watanabei 265	Linjiang City, China	E126°42′00.14″N41°51′10.03″H993m	MF573612	MF573667	MF573722	MF573777
Corydalis watanabei 273	Harbin City, China	E127°33′33.4″ N45°21′13.8″H400m	MF573613	MF573668	MF573723	MF573778
Corydalis yanhusuo 24	Hefei City, China	E117°10′18.88″N31°50′31.21″H232m	MF573614	MF573669	MF573724	MF573779
Corydalis yanhusuo 275	Chuzhou City, China	E117°59′39.41″N32°03′23.15″H90m	MF573615	MF573670	MF573725	MF573780
Corydalis yanhusuo 280	Mingguang City, China	E118°14′02.33″N32°37′29.13″H318m	MF573616	MF573671	MF573726	MF573781
Corydalis yanhusuo 283	Jinhua City, China	E120°27′06.12″N29°03′28.18″H363m	MF573617	MF573672	MF573727	MF573782
Corydalis yanhusuo 289	Nanjing City, China	E118°57′52.10″N32°30′06.05″H137m	MF573618	MF573673	MF573728	MF573783

Table S2: The universal amplification primers used in this study.

**Primer name**	**Length**	**Sequence**	**References/sources**
matK1166	16	GGCTTACTAATGGGAT	Pérez-Gutiérrez et al. 2015 [11]
matK192	20	CGGGTTGCAAMAATAAAGGA	Pérez-Gutiérrez et al. 2015 [11]
psbA	22	GTTATGCATGAACGTAATGCTC	Sang et al. 1997 [23]
trnH	23	CGCGCATGGTGGATTCACAATCC	Tate and Simpson 2003 [24]
trnG	23	GTAGCGGGAATCGAACCCGCATC	Shaw et al. 2005 [25]
trn2G	23	GCGGGTATAGTTTAGTGGTAAAA	Shaw et al. 2005 [25]
rbcL-1F	20	ATGTCACCACAAACAGAAAC	Kress et al. 2005 [26]
rbcL-724R	20	TCGCATGTACCTGCAGTAGC	Kress et al. 2005 [26]
ITS4	20	TCCTCCGCTTATTGATATGC	Baldwin et al. 1992 [27]

ITS5B	22	GGAAGGAGAAGTCGTAACAAGG	Baldwin et al. 1992 [27]
ITS2F	20	ATGCGATACTTGGTGTGAAT	Hou et al. 2013 [28]
ITS3R	21	GACGCTTCTCCAGACTACAAT	Hou et al. 2013 [28]
